# Intraoperative pH Is a Reliable Prognostic Factor for Patients With Periampullary Carcinoma Undergoing Pancreaticoduodenectomy

**DOI:** 10.3389/fonc.2021.764572

**Published:** 2021-11-05

**Authors:** Chao Dang, Min Wang, Tingmei Wang, Renyi Qin

**Affiliations:** ^1^ Department of Pancreatic-Biliary Surgery, Affiliated Tongji Hospital, Tongji Medical College, Huazhong University of Science and Technology, Wuhan, China; ^2^ Department of Dermatology, Affiliated Tongji Hospital, Tongji Medical College, Huazhong University of Science and Technology, Wuhan, China

**Keywords:** pancreaticoduodenectomy, pancreatic cancer, pH, prognostic factor, weakly alkaline

## Abstract

A reliable prognostic factor for periampullary carcinoma is critical to improve surgical outcomes. Intraoperative acidosis reflects the incidence of intraoperative adverse events and impact the prognosis. In this study, 612 patients with periampullary carcinoma who underwent pancreaticoduodenectomy (PD) were divided into high- and low-pH groups according to the cut-off value of receiver operating characteristic curve (7.34). Through statistical analysis of the difference between the high- and low-pH group, it was found that the low-pH group had worse short-term prognosis than the high pH group, and intraoperative pH was an independent prognostic factor for patients with periampullary carcinoma undergoing PD. In addition, patients who underwent laparoscopic pancreaticoduodenectomy had a more alkaline pH after surgery. This is of great help for early judgment of short-term and even long-term prognosis of patients with pancreatic cancer after surgery, and can even guide clinicians to improve prognosis by early adjustment of pH value.

## Introduction

Periampullary adenocarcinoma, which may originate in the pancreas, distal common bile duct, duodenum, or ampulla, is a common malignancy with an increasing incidence in recent years (1). Pancreaticoduodenectomy (PD) is a major abdominal procedure for the treatment of periampullary adenocarcinoma, but it has a high inpatient mortality rate (2, 3). Postoperative complications are potential causes of death after PD (4, 5). Early prediction of postoperative complications and reduction of short-term mortality are important for improving the prognosis of periampullary carcinoma.

A low serum pH is associated with increased complications and mortality after major surgery (6, 7). In fact, intraoperative acidosis reflects the incidence of intraoperative adverse events, and deficient tissue perfusion is an important factor affecting healing. A prolonged duration of intraoperative hypotension, excessive blood loss, excessive blood transfusion, hypothermia, a long operation time, an improper operative technique, inadequate drug administration, excessive fluid administration, or the occurrence of clinically negligible adverse events can lead to intraoperative metabolic acidosis, which reflects inadequate tissue perfusion (8). PD is associated with a long operation time, large amount of blood loss, and risk of hypothermia, resulting in acidosis. However, few studies have addressed the effect of pH on the postoperative complications and prognosis of PD.

Laparoscopic pancreaticoduodenectomy (LPD) is a technology that has gradually matured in recent years. Compared with open pancreaticoduodenectomy (OPD), it has the advantages of less blood loss and faster postoperative recovery (9, 10). However, no reports have addressed the effects of these two surgical methods on intraoperative pH. The present study was performed to evaluate the effects of intraoperative pH on the postoperative complications and short-term prognosis of patients with periampullary carcinoma undergoing PD and to compare the intraoperative pH between LPD and OPD.

## Materials and Methods

### Study Population and Patient Selection

This retrospective study collected 635 periampullary carcinoma patients receiving PD treatment between June 2012 and January 2018, including demographic data, surgical variables, postoperative outcomes, pathological results, and extended follow-up. Twenty-three patients with inadequate baseline data or missing primary outcome data were excluded. A total of 612 patients were included in this study. Data were collected on patient characteristics, surgical details, morbidity and mortality, postoperative hospital stay, and pathological outcomes. Percutaneous transhepatic cholangial drainage (PTCD) were performed for all patients with elevated bilirubin preoperatively, and surgical treatment was performed after the bilirubin returned to normal. Preoperative examination included appropriate imaging diagnosis to exclude distant metastases. Preoperative characteristics included age, sex, complications, body mass index (BMI), and American society of anesthesiologists (ASA) score. Surgical details include operative time (from incision to wound closure), estimated blood loss, and transfusion volume (obtained from anesthesia records). Postoperative follow-up was conducted for 3 months (90 days), and all complications were recorded according to the Clavien-Dindo (CD) system score. Because no objective criteria to recommend laparoscope or open PD in periampullary carcinoma, the surgical method depended on preoperative evaluation and patient’s choice of will. Vascular involvement or prior surgical history or diaphanous variation were taken into account, but were not absolute contraindications to LPD. All patients were informed of the need for surgery and possible complications, as well as the pros and cons of laparoscopic versus open surgery. All patients receiving OPD surgery were performed by 5 experienced doctors in the center, and LPD was performed by Dr. Qin alone. All patients were followed up according to tumor grade to monitor tumor recurrence. The patients were regularly followed up one month after the operation, and then every three months for two years. Follow-up was conducted every six months thereafter. The follow-up of these included patients ended in December 2019. Overall survival (OS) is defined as the time interval between the date of surgery and the date of death.

### Individualized Surgical Strategy

All OPD surgery were performed by 5 experienced surgeons and LPD was mainly performed by one experienced surgeon (Dr. Qin, director of department of Biliary–Pancreatic Surgery). OPD is performed in the supine position following standard Whipple procedures. With regard to LPD, our center for the first time proposed an “individualized surgical strategy” for LPD. Conventional “five-hole method” can meet the needs of surgical operation. Different from the viewpoint of the first assistant and the dominant surgeon advocated by many centers, our center adopts the fixed strategy of the dominant surgeon, which reduces the change of surgical staff by constantly adjusting body position to cooperate with the exposure of surgical field.

According to whether the tumor invaded or compressed the peripheral vascular system and the degree of invasion, we divided the tumors around the head of the pancreas into five types (11), and conducted specific individualized surgical strategies for each type of pancreatic cancer based on surgical experience and other literature. The most commonly used preferred route of the common retroperitoneal artery in our center had been described in detail in previous articles (12). The advantages of this approach are as follows: firstly, the resectability of the tumor was defined; Secondly, through the retroperitoneal route, the root of all the blood supply arteries on the head of the pancreas was preferentially severed, which reduced the amount of blood loss, prevented massive intraoperative bleeding and ensured safety. In addition, the R0 resection rate of carcinoma of the head of the pancreas was increased, and the vascular resection length was reduced. The reconstruction of digestive tract was Child type. For pancreas-intestinal anastomosis, the center uses our own original “implantable anastomosis” which had been described in detail in previous articles (13).

### Definitions

PH was measured by arterial blood drawn by the anesthesiologist after specimen removal was completed during the operation. Operative time was the time from the skin incision or trocar to the complete skin closure. Intraoperative estimated blood loss was recorded the anesthesiologist through a vacuum system. Postoperative hospital stay was defined as the days from surgery to discharge. Morbidity and mortality were defined as any complications or deaths that occurred during hospitalization or within 30 days of discharge after surgery. Readmission within 30 days after surgery was considered unplanned. OS was defined as the time interval between the date of surgery and the date of death. Deaths during hospitalization were excluded in the calculation of postoperative hospital stay. Postoperative complications were evaluated according to the CD classification system (14) and including postoperative pancreatic fistula (15), delayed gastric emptying (16), bile leakage (17), postoperative bleeding (18).

### Statistical Analysis

Continuous variables are reported as mean with standard deviation or median with interquartile range, and categorical data are presented as frequency. Comparisons between two groups were conducted using Student’s t-test or the Mann–Whitney test for quantitative data and the chi-square test or Fisher’s exact test for qualitative data. Survival analysis was performed by the Kaplan–Meier method, and the log-rank test was used to compare the different survival curves. The cut-off values of pH for predicting 18-month survival were determined using a receiver operating characteristic (ROC) curve, and the pH was then dichotomized into low and high groups. Univariate Cox regression analysis was conducted to estimate the risk factors for OS, and variables with a *P* value of <0.1 were then entered into the multivariate Cox regression model. The hazard ratio and 95% confidence interval were calculated, and the lower limit of the 95% confidence interval above zero was considered a significant risk factor. All statistical analyses were performed using SAS statistical software (SAS Institute, Inc., Cary, NC, USA), and two-sided hypothesis testing with a predetermined level of *P* < 0.05 was considered statistically significant.

## Results

### Patient Characteristics and ROC Analysis

**Figure 1:**
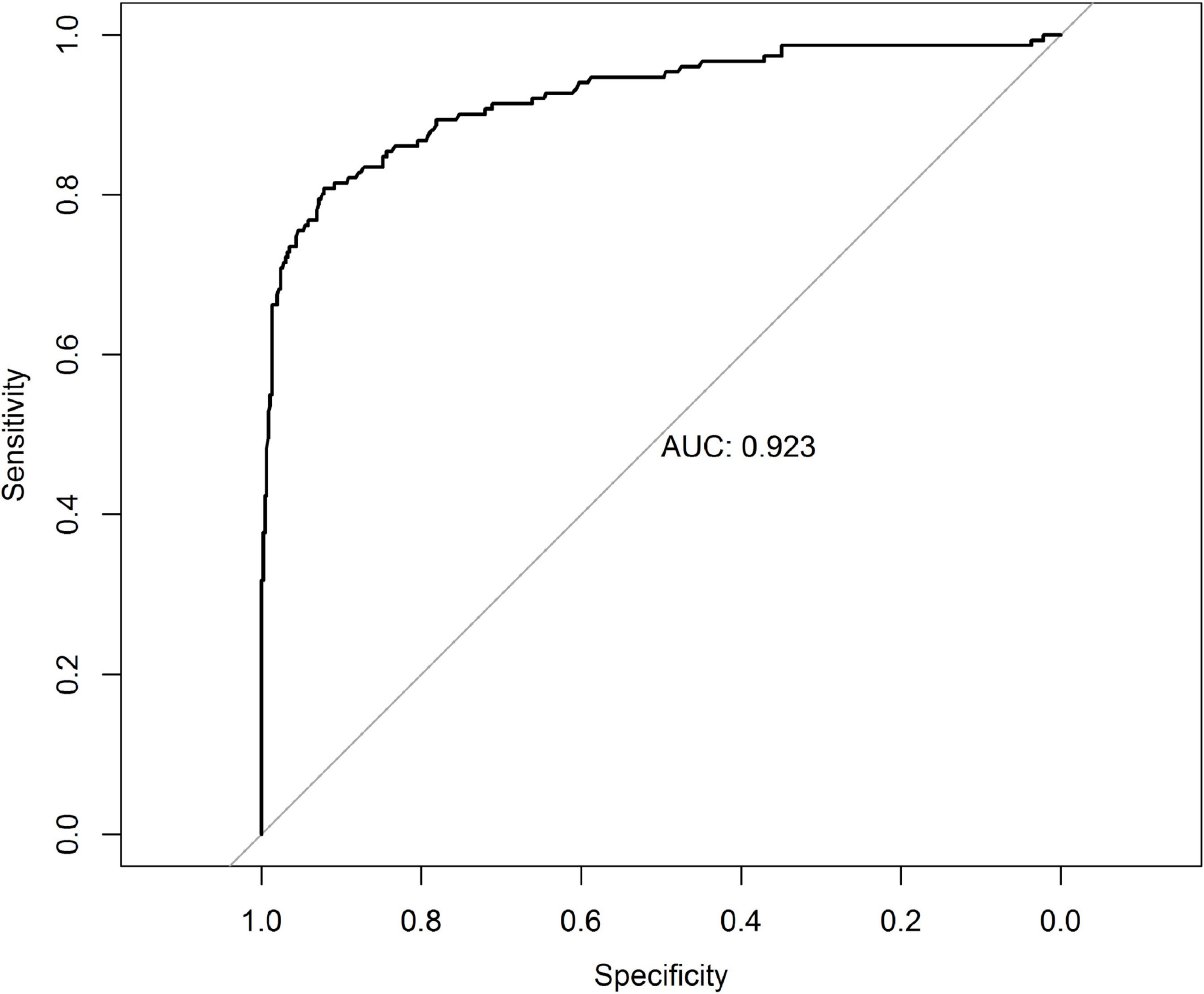
The receiver operating characteristic curve grouped by pH for survival at 18 months after pancreaticoduodenectomy. AUC, area under the curve.

From June 2012 to January 2018, 612 patients with periampullary adenocarcinoma underwent PD in our center. The last follow-up was on 15 December 2019, and the median follow-up period was 18 months (IQR, 6–42). The pH was recorded before suturing peritoneum, and the median pH was 7.37 (range, 6.69–7.64). As shown in **Figure 1**, the patients were divided into a high and low pH group according to the cut-off value of the ROC curve (7.34) (a sensitivity of 80.8% and a specificity of 92.2%). The area under the curve (AUC) of pH was 0.923 (95% CI, 0.897–0.952, *P* < 0.001). The low pH group (pH ≤ 7.34) comprised 421 patients, and the high pH group (pH >7.34) comprised 191 patients.

### Correlations Between pH and Patient Characteristics

The relationships between the pH and the patients’ clinical parameters are shown in **Table 1**. There was no significant correlation between pH and age, BMI, sex, blood group, diabetes mellitus, family history, tumor size, alkaline phosphatase, gamma-glutamyl transpeptidase, cholesterol, triglyceride, lactate dehydrogenase, total protein, albumin, white blood cells, preoperative total bilirubin, preoperative biliary drainage, ASA, TNM stage, histopathological diagnosis, or serum tumor markers.

**Table 1:** Baseline characteristics of the patients in Low-pH and High-pH group.

Variable	Low-pH	High-pH	P value
	(421)	(191)	
Age, Mean (SD), year	55.89 (9.94)	54.20 (11.70)	0.066
BMI, Mean (SD), kg/m^2^	21.93 (3.09)	21.80 (2.76)	0.630
Sex, N (%)			0.128
Males	268 (63.66)	115 (60.21)	
Females	153 (36.34)	76 (39.80)	
Blood group, N (%)			0.323
A	150 (35.63)	54 (28.42)	
B	103 (24.47)	54 (28.42)	
AB	30 (7.13)	17 (8.95)	
O	138 (32.78)	65 (34.21)	
Diabetes mellitus, N (%)			0.320
No	395 (94.27)	176 (92.15)	
Yes	24 (5.73)	15 (7.85)	
Family history, N (%)			0.439
No	412 (98.10)	189 (98.95)	
Yes	8 (1.90)	2 (1.05)	
Tumor size, Mean (SD), cm	2.70 (1.70)	2.65 (2.38)	0.782
ALP, Mean (SD), U/L	299.40 (278.11)	277.99 (280.77)	0.382
r-GT, Mean (SD), U/L	419.39 (459.69)	393.98 (481.88)	0.535
Cholesterol, Mean (SD), mmol/L	5.02 (2.01)	4.74 (1.76)	0.100
Triglyceride, Mean (SD), mmol/L	1.93 (1.64)	1.75 (1.33)	0.301
LDH, Mean (SD), U/L	188.55 (57.78)	191.59 (62.64)	0.640
Total protein, Mean (SD), g/L	67.02 (8.17)	67.00 (6.25)	0.975
Albumin, Mean (SD), g/L	37.86 (5.04)	38.41 (4.27)	0.199
White Blood Cell, Mean (SD),10^9^/L	6.17 (2.43)	5.97 (4.85)	0.487
Preoperative total bilirubin, Mean (SD), μmol	69.95 (39.61)	64.71 (37.77)	0.375
Preoperative biliary drainage, N (%)			0.682
No	275 (65.32)	128 (67.02)	
Yes	146 (34.68)	63 (32.98)	
ASA, N (%)			0.088
>II	58 (13.78)	17 (8.90)	
≤II	363 (86.22)	174 (91.10)	
Depth of tumor, N (%)			0.200
T1	176 (41.81)	97 (50.79)	
T2	197 (46.79)	80 (41.88)	
T3	48 (11.40)	14 (7.33)	
Lymph node metastasis, N (%)			0.390
N0	296 (70.37)	129 (67.54)	
N1	107 (25.31)	48 (25.13)	
N2	18 (4.32)	14 (7.33)	
Distance metastasis, N (%)			0.921
M0	421 (100)	191 (100)	
M1	0 (0)	0 (0)	
pStage, N (%)			0.294
IA	136 (32.30)	63 (32.98)	
IB	118 (28.03)	58 (30.37)	
IIA	38 (9.03)	6 (3.14)	
IIB	107 (25.42)	48 (25.13)	
III	22 (5.23)	16 (8.38)	
IV	0 (0)	0 (0)	
Histopathological diagnosis, N (%)			0.357
Bile duct carcinoma	38 (9.03)	16 (8.38)	
Ampullary carcinoma	41 (9.74)	19 (9.95)	
Pancreatic carcinoma	180 (42.76)	83 (43.46)	
Duodenal carcinoma	162 (38.48)	73 (38.22)	
CA19-9, Mean (SD), u/ml	490.34 (1523.39)	309.16 (1196.47)	0.155
CA125, Mean (SD), u/ml	23.55 (30.31)	22.89 (35.64)	0.851
CEA, Mean (SD), ng/ml	6.56 (40.82)	4.11 (9.76)	0.422

BMI, body mass index; ALP, alkaline phosphatase; r-GT, gamma-glutamyl transpeptidase; LDH, lactate dehydrogenase; ASA, American society of anesthesiologists; CA19-9, carcinoembryonic antigen 19-9; CA125, carcinoembryonic antigen 125; CEA, carcinoembryonic antigen; SD, standard derivation; IQR, interquartile range.

### Correlations Between pH and Intraoperative Characteristics

As shown in **Table 2**, low pH was associated with a longer operative time (median, 363 *vs* 307 min; *P* < 0.001), a higher intraoperative bleeding volume (mean, 445 *vs* 259 *ml*; *P* < 0.001), and a higher intraoperative blood transfusion volume (mean, 1.62 *vs* 0.98 U; *P* = 0.015). There was no significant difference in the R0 removal rate between the low and high pH groups (median, 84.56% *vs* 87.43%; *P* = 0.350). Similarly, there was no significant difference in the number of lymph nodes cleared between the low and high pH groups (*P* = 0.476). There was a significant difference in the operation method between the low and high pH groups (*P* < 0.001).

**Table 2:** Comparison of intraoperative factors between Low-pH and High-pH group.

Variable	Low-pH	High-pH	P value
	(421)	(191)	
Duration of surgery, Mean (SD), min	362.98 (111.20)	307.43 (86.81)	<0.001
Intraoperative bleeding, Mean (SD), ml	444.52 (476.63)	259.38 (327.14)	<0.001
Red blood cell transfusion, Mean (SD), U	1.62 (2.44)	0.98 (3.94)	0.015
Operation method, N (%)			<0.001
OPD	308 (73.16)	54 (28.27)	
LPD	113 (26.84)	137 (71.73)	
R state, N (%)			0.350
R0	356 (84.56)	167 (87.43)	
R1	65 (15.44)	24 (12.57)	
Lymph node dissection, Median (IQR)	15 (13~25)	16 (13~28)	0.476

LPD, laparoscopic pancreaticoduodenectomy; OPD, open pancreaticoduodenectomy; SD, standard derivation; IQR, interquartile range.

### Correlations Between pH and Postoperative Characteristics

The patients’ postoperative factors are shown in **Table 3**. The rate of total complications was significantly higher in the low pH group than high pH group (31.83% *vs* 18.32%, *P* = 0.026). Specifically, postoperative pancreatic fistula (19.95% *vs* 10.99%, *P* = 0.031), renal failure (2.14% *vs* 0.00%, *P* = 0.016), infection (5.24% *vs* 1.05%, *P* = 0.021), and delayed gastric emptying (26.37% *vs* 17.28%, *P* = 0.024) were more common in the low pH group. There was no significant difference in the rate of positive lymph nodes sent for examination between the low and high pH groups (*P* = 0.385). There was also no difference in the 30-day unplanned readmission rate after surgery (4.75% *vs* 4.71%, *P* = 0.163); however, the low pH group had a longer postoperative hospital stay (23 *vs* 19 days, *P* = 0.043). The reoperation rate was similar between the low and high pH groups (2.14% *vs* 0.52%, *P* = 0.144). The 90-day mortality rate was significantly higher in the low than high pH group (4.99% *vs* 0.52%, *P* < 0.001).

**Table 3:** Comparison of short-term postoperative results between Low-pH and High-pH group.

Variable	Low-pH	High-pH	P value
	(421)	(191)	
Postoperative hospital stay, Mean (SD), day	22.94 (9.73)	19.14 (10.11)	0.043
Positive lymph node, Median (IQR)	0.82 (2.34)	0.66 (1.59)	0.385
Aggregate complications, N (%)	134 (31.83)	35 (18.32)	0.026
Renal failure	9 (2.14)	0 (0.00)	0.016
Pulmonary complications	4 (0.95)	1 (0.52)	0.449
Hepatic failure	2 (0.48)	0 (0.00)	0.340
Gastrointestinal fistula	0 (0.00)	0 (0.00)	0.602
Biliary leakage	2 (0.48)	3 (1.57)	0.164
Postpancreatectomy hemorrhage	33 (7.86)	16 (8.38)	0.827
Pancreatic fistula	84 (19.95)	21 (10.99)	0.031
Delayed gastric emptying of grade B/C	111 (26.37)	33 (17.28)	0.024
Infection	22 (5.24)	2 (1.05)	0.021
Reoperation N (%)	9 (2.14)	1 (0.52)	0.144
30 days unplanned readmission, N (%)	20 (4.75)	9 (4.71)	0.983
90-Day mortality, N (%)	21 (4.99)	1 (0.52)	0.006

SD, standard derivation; IQR, interquartile range.

### pH Is an Independent Prognostic Marker for Patients Undergoing PD

**Figure 2:**
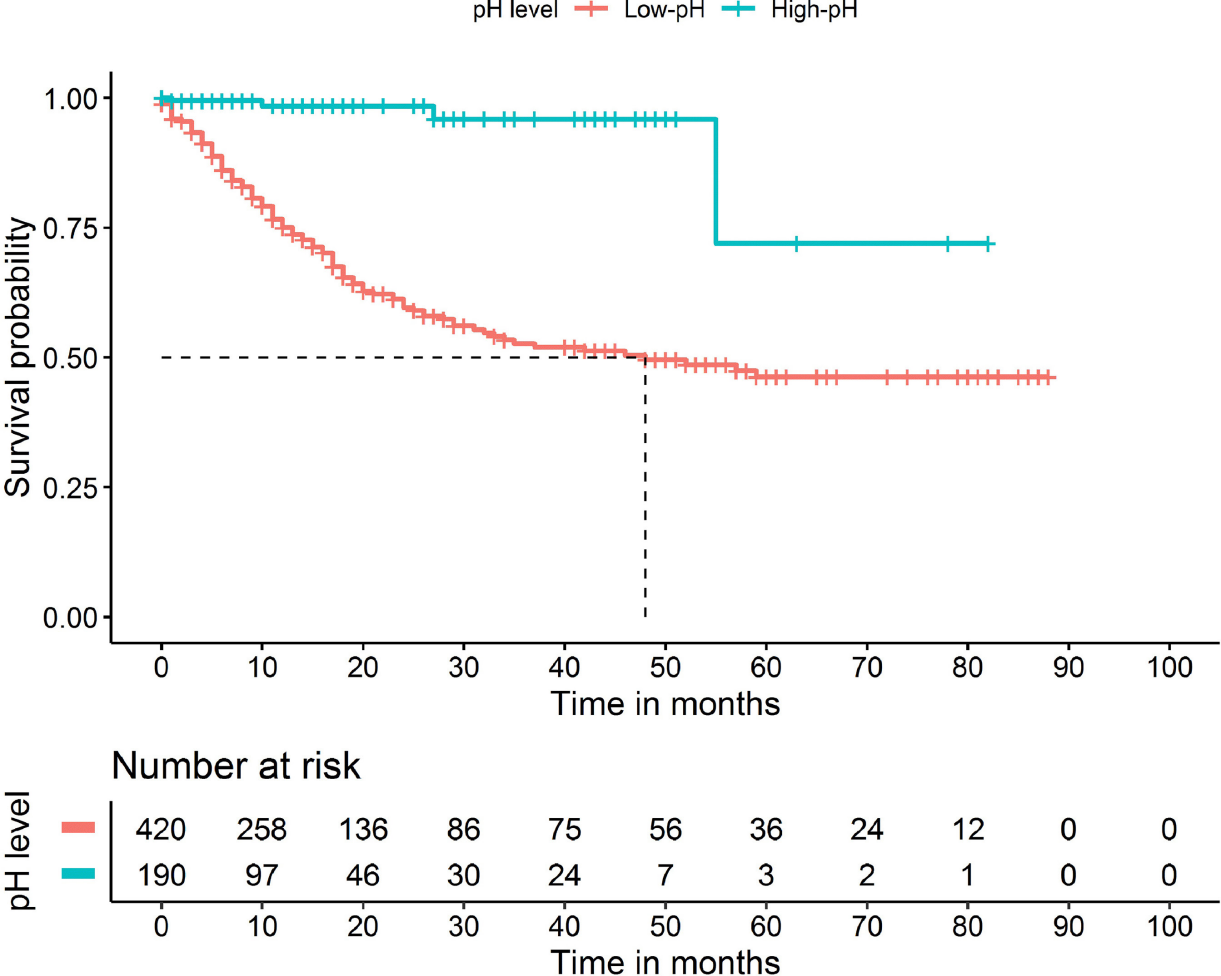
Kaplan-Meier curves for overall survival of patients who received pancreaticoduodenectomy based on pH (P <0.001).

As shown in **Table 4**, we performed univariate and multivariate survival analyses for OS to determine whether pH was an independent prognostic factor for periampullary carcinoma. Single-factor analysis showed that pH, operation method, ASA, tumor stage, R state, and pancreas texture were significantly correlated with OS (*P* < 0.001). To determine the independent prognostic factors, the important factors in the univariate analysis were included in the multivariate analysis. The multivariate analysis showed that pH, R state and tumor stage were independent prognostic factors for OS (*P* < 0.001). Besides, the Kaplan–Meier survival curve suggested that low-pH was associated with low OS, and the difference in survival rate between the low-pH group and the high-pH group was statistically significant (*P* < 0.001, **Figure 2**).

**Table 4:** Risk factors analysis of pH as a prognostic factor for periampullary carcinoma.

Parameter	Univariate Analysis		Multivariate Analysis	
	HR (95%CI)	P Value	HR (95%CI)	P Value
PH				
≤7.43	Reference		Reference	
>7.43	0.074 (0.028, 0.201)	<0.001	0.111 (0.040~0.306)	<0.001
PD				
No	Reference		Reference	
LPD	0.589 (0.410~0.845)	0.004	1.418 (0.974~2.063)	0.068
Gender				
Males	Reference			
Females	0.934 (0.673~1.296)	0.682		
Blood type				
A	Reference			
B	0.951 (0.617~1.465)	0.819		
AB	1.852 (1.092~3.142)	0.222		
O	1.038 (0.694~1.553)	0.857		
Diabetes mellitus				
No	Reference			
Yes	0.579 (0.237~1.412)	0.230		
Family history				
No	Reference			
Yes	0.874 (0.323~2.367)	0.792		
History of surgery				
No	Reference			
Yes	1.380 (0.992~1.919)	0.056		
ASA				
>II	Reference		Reference	
≤II	0.635 (0.413~0.976)	0.039	0.766 (0.480~1.222)	0.264
pStage, N (%)				
IA	Reference		Reference	
IB	2.122 (1.287~3.497)	0.003	2.715 (1.495~4.931)	0.001
IIA	2.708 (1.377~5.322)	0.004	3.741 (1.332~10.502)	0.012
IIB	3.082 (1.874~5.069)	<0.001	3.020 (1.184~7.703)	0.021
III	2.632 (1.132~6.116)	0.025	3.220 (0.539~9.146)	0.027
IV	2.790 (0.657~11.850)	0.164	3.255 (0.724~14.629)	0.024
Preoperative biliary drainage				
No	Reference			
Yes	1.331 (0.953~1.861)	0.094		
R state				
R0	Reference		Reference	
R1	2.129 (1.483~3.056)	<0.001	1.568 (0.982~2.505)	0.060
Pancreas texture				
Soft	Reference		Reference	
Hard	1.860 (1.292~2.679)	0.001	1.366 (0.889~2.099)	0.155
Moderate	1.340 (0.860~2.089)	0.196	1.305 (0.802~2.126)	0.284

ASA, American society of anesthesiologists; SD, standard derivation; IQR, interquartile range.

### Differences in Effect of LPD and OPD on pH

As shown in **Table 5**, the average pH in the LPD group was 7.41, which was significantly higher than that in the OPD group (7.34) (*P* < 0.001). The operation time was significantly shorter in the LPD than OPD group (305 *vs* 374 min, *P* < 0.001), as was the blood loss volume (205 *vs* 512 ml, *P* < 0.001). The intraoperative red blood cell infusion volume was also significantly lower in the LPD than OPD group (0.42 *vs* 2.12 U, *P* < 0.001). The postoperative hospital stay was significantly shorter in the LPD than OPD group (20 *vs* 21 days, *P* = 0.005). There were no statistically significant differences in postoperative complications, 90-day mortality, 30-day unplanned hospitalization, or the R0 resection rate between the two groups.

**Table 5:** Comparison characteristics between laparoscopic pancreaticoduodenectomy (LPD) and open pancreaticoduodenectomy (OPD).

Parameter	OPD	LPD	P value
pH Mean (std)	7.34 (0.11)	7.41 (0.12)	<0.001
Age, Mean (SD), year	54.94 (10.42)	55.97 (10.71)	0.234
BMI Mean (SD), kg/m^2^	22.08 (3.24)	21.64 (2.61)	0.086
Sex (Males), N (%)	215 (59.39)	145 (58.00)	0.731
Diabetes mellitus (Yes), N (%)	25 (6.94)	14 (5.60)	0.504
Family history (Yes), N (%)	9 (2.49)	1 (0.40)	0.045
History of surgery (Yes), N (%)	130 (36.01)	78 (31.20)	0.217
CA19-9, Median Mean (SD), u/ml	482.62 (1478.93)	363.03 (1357.31)	0.320
CA125, Median Mean (SD), u/ml	23.43 (31.14)	23.18 (33.59)	0.938
CEA, Median Mean (SD), ng/ml	6.83 (43.68)	4.30 (10.84)	0.381
Preoperative total bilirubin Median Mean (SD), μmol	72.50 (43.49)	63.60 (32.77)	0.101
Duration of surgery, median Mean (SD), min	374.01 (109.50)	304.62 (89.39)	<0.001
Intraoperative bleeding, median Mean (SD), ml	512.32 (516.50)	204.89 (200.13)	<0.001
ASA, N (%)			0.096
>II	51 (14.09)	24 (9.60)	
≤II	311 (85.91)	226 (90.40)	
pStage, N (%)			0.190
IA	113 (31.21)	86 (34.40)	
IB	102 (28.18)	74 (29.60)	
IIA	27 (7.46)	17 (6.80)	
IIB	98 (27.07)	57 (22.80)	
III	22 (6.08)	16 (6.40)	
IV	0 (0)	0 (0)	
R state, N (%)			0.138
R0	303 (83.70)	220 (88.00)	
R1	59 (16.30)	30 (12.00)	
Red blood cell transfusion, Mean (SD), U	2.12 (3.63)	0.42 (1.16)	<0.001
Lymph node dissection, median (IQR)	7.31 (6.42)	6.68 (6.21)	0.236
Positive lymph node, Median (IQR)	0.84 (2.42)	0.67 (1.62)	0.340
Postoperative hospital stay, median (IQR), day	21 (17~28)	20 (16~25)	0.005
30 days unplanned readmission, N (%)	14 (3.87)	15 (6.00)	0.222
Aggregate complications (Yes), N (%)	102 (28.18)	67 (26.80)	0.708
Reoperation (Yes) N (%)	6 (1.66)	4 (1.60)	0.956
90-Day mortality, N (%)	14 (3.87)	8 (3.20)	0.663

BMI, body mass index; CA19-9, carcinoembryonic antigen 19-9; CA125, carcinoembryonic antigen 125; CEA, carcinoembryonic antigen; SD, standard derivation; IQR, interquartile range.

## Discussion

The annual incidence of periampullary adenocarcinoma has been steadily increasing. The only effective treatment for periampullary adenocarcinoma is PD, but the cure rate is only 20% (19). PD is a complicated operation, and its numerous postoperative complications and low survival rate are of concern among surgeons (20–22). Therefore, many institutions have studied the factors affecting the postoperative prognosis of patients undergoing PD. Because of the long operation time and high degree of difficulty of PD, clinical adverse events such as hypotension, excessive blood loss, and hypothermia can lead to postoperative acidosis. In the present study, we investigated the relationship of intraoperative pH with postoperative complications and survival. To our knowledge, this is the first study to investigate the effect of intraoperative pH on the prognosis underwent PD.

Using ROC curve analysis, the patients were divided into a high and low pH group according to the cut-off value of 7.34 for 18-month survival. The results showed that intraoperative pH was not influenced by preoperative factors. However, an analysis of intraoperative factors showed that the low pH group contained more patients who lost a larger amount of blood during surgery or had a longer operation time. This result is consistent with the view that the intraoperative pH changes in accordance with a prolonged operation time and increased intraoperative blood loss (8). For patients undergoing PD, both OPD and LPD are used as routine operations in our center. Our statistical analysis also showed that the high pH group contained a greater proportion of patients undergoing LPD than OPD. Our analysis of the patients’ postoperative characteristics showed that a lower pH was associated with a higher incidence of postoperative total complications. In particular, the incidence of pancreatic leakage was higher in the low pH group; the patients in this group also had longer hospital stays and higher 90-day mortality. These findings indicate that a low intraoperative pH is associated with a poor short-term prognosis. Therefore, when the pH of patients with periampullary cancer is <7.34 during surgical procedures, appropriate measures should be taken as soon as possible to increase the pH. The prognosis after pH improvement is not discussed in this paper; therefore, the efficacy of pH improvement remains to be further studied.

In the present study, we also evaluated the risk factors for postoperative survival of patients with periampullary carcinoma underwent PD and found that intraoperative pH was an important factor affecting the prognosis. Kaplan–Meier analysis clearly showed significant differences in survival of patients with periampullary carcinoma between the low and high pH groups. Because of the short follow-up time, the effect of pH on the long-term prognosis needs to be further verified. The mechanism by which intraoperative pH can affect the prognosis of periampullary carcinoma is still unclear. Indeed, whether acidosis is a marker of postoperative complications or the cause of postoperative complications remains unknown (23). Although there are many causes of acidosis, such as a large intraoperative blood loss volume, long operation time, hypothermia, improper surgical technique, and others, many of these factors can be avoided, and this is the focus of our attention. Because of the difficult operation, long operation time, and large intraoperative blood loss associated with PD, perioperative management is particularly important to avoid low intraoperative pH. On this basis, it is especially important to treat low pH early in the perioperative period because it may help to avoid complications such as infection or kidney damage, which almost certainly lead to death. Evidence suggests that acidosis may interfere with hemodynamics (24) and innate immunity (25), which may help to explain the high complication and short-term mortality rates associated with a low pH in this study; however, the exact mechanism remains unclear and needs further exploration. Although different types of acidosis may have different effects on the prognosis (26), we did not further classify acidosis. However, intraoperative pH can be an early indicator of a higher risk of postoperative complications and short-term death in patients with periampullary carcinoma, enabling early initiation of treatment.

In recent years, LPD has attracted increasingly more attention. Since 2014, LPD has been widely carried out in our center as a routine surgical method. However, the advantages and disadvantages of LPD and OPD are still controversial. The pH can reflect intraoperative blood loss, body temperature, and other comprehensive conditions; thus, we also wanted to compare the difference in pH between LPD and OPD. We found that the intraoperative pH was significantly higher in the LPD than OPD group, which was closer to the pH value obtained through ROC curve analysis. The reason may be that the operation time was slightly shorter and the intraoperative blood loss was significantly lower for LPD than OPD, which is consistent with the pH reflecting intraoperative blood loss. Compared with OPD, the shorter operative time of LPD is slightly different from that reported by other centers (27); this may be due to the fact that LPD requires a smaller incision and a shorter time to close the abdominal cavity. Furthermore, the laparoscopic pancreaticoduodenectomy technique developed by Professor Qin (13) at our center shortened the operating time. The postoperative hospitalization duration was also shorter in the LPD than OPD group. Thus, patients with a more alkaline pH in the LPD group recovered more quickly and were more likely to leave the hospital sooner, thus entering the next treatment stage.

Our study has many important clinical implications. In particular, this new and innovative study is the first to clearly indicate that a weakly alkaline intraoperative pH affects the prognosis of patients with periampullary carcinoma undergoing PD. However, our study also has some limitations. Because of the observational design and retrospective nature of the study, we were unable to establish a causal relationship between acidosis and prognosis. Additionally, previous studies have shown that different types of acidosis have different clinical consequences, and our study focused only on the pH and did not classify acidosis.

## Conclusions

In summary, the results of this study indicate that the intraoperative pH has a good predictive effect on the postoperative complications and short-term prognosis of patients with periampullary carcinoma undergoing PD. Our risk factor analysis showed that pH can be used as a prognostic factor for periampullary carcinoma. In addition, the faster postoperative recovery after LPD than OPD may be related to less pH reduction during the operation. Considering that this was only a single-center study, a larger number of well-designed prospective studies should be conducted to further confirm this conclusion.

## Data Availability Statement

The original contributions presented in the study are included in the article/supplementary material. Further inquiries can be directed to the corresponding authors.

## Author Contributions

CD: Conceptualization, methodology, formal analysis, investigation, and writing – original draft. MW: Funding acquisition and supervision. TW: Formal analysis. RQ: Funding acquisition and supervision. All authors contributed to the article and approved the submitted version.

## Conflict of Interest

The authors declare that the research was conducted in the absence of any commercial or financial relationships that could be construed as a potential conflict of interest.

## Publisher’s Note

All claims expressed in this article are solely those of the authors and do not necessarily represent those of their affiliated organizations, or those of the publisher, the editors and the reviewers. Any product that may be evaluated in this article, or claim that may be made by its manufacturer, is not guaranteed or endorsed by the publisher.
